# Gastrointestinal Bleeding in Patients with Hereditary Hemorrhagic Telangiectasia: Risk Factors and Endoscopic Findings

**DOI:** 10.3390/jcm9010082

**Published:** 2019-12-28

**Authors:** José María Mora-Luján, Adriana Iriarte, Esther Alba, Miguel Ángel Sánchez-Corral, Ana Berrozpe, Pau Cerdà, Francesc Cruellas, Jesús Ribas, Jose Castellote, Antoni Riera-Mestre

**Affiliations:** 1 HHT Unit, Hospital Universitari de Bellvitge, 08907 Barcelona, Spain; jmora@bellvitgehospital.cat (J.M.M.-L.); adriana.iriarte@bellvitgehospital.cat (A.I.); estheralba@bellvitgehospital.cat (E.A.); mmena@bellvitgehospital.cat (M.Á.S.-C.); pau.pauet@gmail.com (P.C.); fcruellas@bellvitgehospital.cat (F.C.); jribass@bellvitgehospital.cat (J.R.); jcastellote@bellvitgehospital.cat (J.C.); 2 Internal Medicine Department, Hospital Universitari de Bellvitge, 08907 Barcelona, Spain; 3 Bellvitge Biomedical Research Institute (IDIBELL), L’Hospitalet de Llobregat, 08907 Barcelona, Spain; aberrozpe@bellvitgehospital.cat; 4 Radiology Department, Hospital Universitari de Bellvitge, 08907 Barcelona, Spain; 5 Cardiology Department, Hospital Universitari de Bellvitge, 08907 Barcelona, Spain; 6 Gastroenterolgy Department, Hospital Universitari de Bellvitge, 08907 Barcelona, Spain; 7 Otorhinolaringology Department, Hospital Universitari de Bellvitge, 08907 Barcelona, Spain; 8 Pneumology Department, Hospital Universitari de Bellvitge, 08907 Barcelona, Spain; 9 Clinical Sciences Department, Faculty of Medicine and Health Sciences, Universitat de Barcelona, L’Hospitalet de Llobregat, 08907 Barcelona, Spain

**Keywords:** hereditary hemorrhagic telangiectasia, rare diseases, telangiectasis, transforming growth factor-beta (TGF-β), Smad pathway, gastrointestinal bleeding

## Abstract

Background: We aimed to describe risk factors for gastrointestinal (GI) bleeding and endoscopic findings in patients with hereditary hemorrhagic telangiectasia (HHT). Methods: This is a prospective study from a referral HHT unit. Endoscopic tests were performed when there was suspicion of GI bleeding, and patients were divided as follows: with, without, and with unsuspected GI involvement. Results: 67 (27.9%) patients with, 28 (11.7%) patients without, and 145 (60.4%) with unsuspected GI involvement were included. Age, tobacco use, endoglin (ENG) mutation, and hemoglobin were associated with GI involvement. Telangiectases were mostly in the stomach and duodenum, but 18.5% of patients with normal esophagogastroduodenoscopy (EGD) had GI involvement in video capsule endoscopy (VCE). Telangiectases ≤ 3 mm and ≤10 per location were most common. Among patients with GI disease, those with hemoglobin < 8 g/dL or transfusion requirements (65.7%) were older and had higher epistaxis severity score (ESS) and larger telangiectases (>3 mm). After a mean follow-up of 34.2 months, patients with GI involvement required more transfusions and more emergency department and hospital admissions, with no differences in mortality. Conclusions: Risk factors for GI involvement have been identified. Patients with GI involvement and severe anemia had larger telangiectases and higher ESS. VCE should be considered in patients with suspicion of GI bleeding, even if EGD is normal.

## 1. Introduction

Hereditary hemorrhagic telangiectasia (HHT) or Rendu-Osler-Weber syndrome (ORPHA774) is an autosomal dominant rare vascular disease characterized by telangiectases and larger vascular malformations (VMs) [1,2,3]. HHT can be diagnosed either clinically using the Curaçao criteria (recurrent epistaxis, muco-cutaneous telangiectasia, visceral lesions, and family history) or through a molecular gene test [4,5,6]. Mutations in endoglin (*ENG*) and activin A receptor type II-like 1 (*ACVRL1*) genes are detected in approximately 90% of cases submitted to molecular diagnosis for clinical suspicion of HHT and cause HHT1 and HHT2, respectively [2,7,8,9]. Mutations in *SMAD4* (encoding the transcription factor Smad4) have been described in less than 2% of the HHT population [8]. Endoglin (encoded by *ENG*) is an auxiliary co-receptor at the endothelial cell surface that promotes BMP9 signaling through the activin receptor-like kinase 1 (ALK1; encoded by *ACVRL1*) [2]. Both proteins contribute to the signaling hub formed by BMP9-endoglin-ALK1-Smad with a high impact in the angiogenesis process [10].

Telangiectasis is the characteristic lesion in HHT and shows dilated post capillary venules directly connected with dilated arterioles losing the capillary bed [11]. These dilated microvessels are more prone to hemorrhage due to fragile walls and turbulent blood flow, especially those located in mucosae, such as nasal or gastrointestinal (GI). Telangiectasia in nasal mucosae can cause spontaneous, recurrent epistaxis, usually the earliest and most common clinical manifestation of HHT [2,9,12].

Unlike epistaxis or visceral involvement, which occur since adolescence, GI bleeding begins in the fifth or sixth decades of life [4,13]. The prevalence of GI telangiectasia ranges from 13% to 30% in the overall HHT population to more than 90% in HHT patients with anemia [14,15,16,17,18,19,20]. In HHT patients, GI bleeding is usually chronic, low degree, and in an intermittent fashion [16,17]. However, clinical presentation can be diverse, with some patients presenting none or mild anemia, while others require periodic transfusions. Although argon plasma coagulation (APC) is the most effective endoscopic therapy for active GI bleeding, some patients have either multiple or non-accessible telangiectases for APC and usually require additional or alternative therapies [4,13,16,21]. Therefore, pharmacological treatment, such as with estrogen/progesterone, somatostatin analog octreotide, and bevacizumab, has been considered in these patients. However, these pharmacological therapies have not been well established and are based on either individual case reports or small case series [4,13,16,21,22].

Though GI telangiectasia is included in the visceral lesions defined in the Curaçao criteria, international guidelines only recommend endoscopic study in adult patients with disproportionate anemia to the severity of epistaxis [4,5]. Unfortunately, there is no clear definition for disproportionate anemia, so indication for endoscopic study often depends on physician experience. Though some studies have suggested an association between age or female gender and GI disease, risk factors predisposing GI disease are currently unknown [4,8,13,15,16]. Identifying possible risk factors associated with GI involvement could be useful for HHT clinical management. The objective of the present study is to describe risk factors for GI involvement and to assess endoscopic findings and outcomes in this scenario.

## 2. Materials and Methods

### 2.1. Study Design

This is a prospective, observational study, which includes all consecutive patients attended in a referral HHT unit in a university hospital from September 2011 to June 2019. This HHT unit attends adult patients from all over Catalonia (Spain), which accounts for seven million inhabitants. During this period, a total of 330 patients were visited in our unit. All patients provided oral consent for participation in the study in accordance with local Ethic Committee requirements. Patients with a definite diagnosis according to the Curaçao criteria (meeting ≥ 3 criteria) or a positive genetic study were included [4,5,6]. We followed the Strengthening the Reporting of Observational Studies in Epidemiology (STROBE) statement guidelines for observational cohort studies [23].

The main objective was to describe risk factors for GI involvement among patients with definite or molecular diagnosis of HHT. Secondary objectives were to compare endoscopic findings among patients with GI involvement according to clinical severity and to assess clinical outcomes according to GI involvement.

### 2.2. Assessment of Gastrointestinal Involvement

Suggestive symptoms/signs of GI involvement were defined as either the presence of overt upper or lower GI bleeding or the presence of anemia that is disproportionate to the severity of epistaxis [4]. Endoscopic study (GIF-Q165; Olympus, Hamburg, Germany) was performed when suggestive symptoms/signs of GI involvement were present. In patients with active or recently bleeding telangiectases, treatment with APC was performed. A video capsule endoscopy (VCE) (PillCamSB 3; Medtronic, Yokneam, Israel) study was mostly performed in patients with persistent anemia after APC treatment.

Endoscopic study was defined as positive when HHT suggestive telangiectasia was found in the GI tract. Normal endoscopic study or non-suggestive findings of HHT were defined as a negative study. Using these criteria and according to GI disease, three groups were established: (a) GI involvement: patients with positive endoscopic study; (b) no GI involvement: patients with negative endoscopic study; (c) unsuspected GI involvement: patients without suggestive symptoms/signs of GI bleeding and no endoscopic study performed.

Among patients with GI involvement, telangiectases were classified according to their number (few: ≤10 telangiectases or multiple: >10 telangiectases) and size (small: ≤3 mm or large: >3 mm) [13]. Patients with GI involvement were classified into two subgroups according to clinical severity: patients with either hemoglobin levels less than 8 g/dL or red blood cell (RBC) transfusion requirements and patients with none of these conditions. Endoscopic findings were compared between both subgroups.

### 2.3. Clinical Variables, Screening Tests, and Follow-Up

Baseline demographic characteristics, comorbidities, history of alcohol or tobacco use (previous or currently), hemoglobin levels, genetic study, blood test, and epistaxis severity score (ESS) were collected. ESS is an online tool that quantifies the epistaxis severity considering different parameters during the previous three months [24].

To screen for pulmonary visceral involvement, a contrast transthoracic echocardiography (TTE) was performed [4,25]. The Barzilai scale was used to establish the degree of right-left shunt (R-L) and the need to undergo a thoracic computed tomography (CT) angiography to confirm the presence of pulmonary arteriovenous (AV) fistula [4,26]. In addition, an abdominal CT angiography was performed to study hepatic and/or abdominal VMs. A cerebral CT angiography or brain MRI angiography was carried out in cases of neurological symptoms or a family history of neurological involvement [4,25].

All patients were prospectively followed-up depending on disease severity, at the treating clinician’s discretion. The lowest hemoglobin level detected and different treatment strategies used during follow-up were recorded. Patients with objectively confirmed GI involvement and severe anemia despite iron therapy and/or requiring blood transfusions were assessed for treatment with octreotide or bevacizumab. The number of packed RBCs transfused before and during both treatments was recorded. Moreover, the need for visits to the emergency department (ED) and/or hospital admissions, the number of RBC transfusions required, and any mortality during follow-up were also registered. These outcomes were compared between HHT groups according to GI involvement.

### 2.4. Statistical Analysis

A descriptive statistical analysis was performed for all categorical and continuous variables and expressed as proportions or means with standard deviations (SD), respectively. Categorical variables were compared with the chi-square test or Fisher’s exact test, whereas the t-test or the Mann–Whitney U test were used to compare continuous variables.

Logistic regression analyses were performed to identify associated risk factors for GI disease and presented as odds ratios (OR) with 95% confidence intervals (95% CI). Logistic regression was performed in patients with suggestive symptoms/signs of GI involvement. For the manual backward stepwise multivariable logistic regression model, we assessed variables that had a significant level of P less than 0.1 in univariable analyses. To analyze the predictive power of the associated risk factors, a receiver operating characteristic (ROC) curve was performed and the area under the curve (AUC) was calculated. *p* values of <0.05 were considered to be statistically significant. Analyses were performed using SPSS, version 15 for the PC (SPSS Inc., Chicago, IL, USA).

## 3. Results

### 3.1. Clinical Characteristics

During the study period, 240 patients met the inclusion criteria. Most patients (57.1%) were female, and mean age was 53.6 ± 13.6 years. Clinical diagnosis of HHT was definitive according to the Curaçao criteria (meeting ≥ 3 criteria) in 229 (95.4%) patients and by a positive molecular test in the remaining 11 (4.6%) patients. A genetic test was carried out in 161 patients: 75 (45.2%) had *ENG* mutation, and 73 (43.9%) had *ACVRL1* mutation, with no mutation found in 13 (7.8%). After screening tests, 176 patients (73.3%) had visceral involvement: 67 (27.9%) with pulmonary AV fistula, 110 (45.8%) with hepatic VMs, 73 (30.4%) with other intraabdominal VM, and 10 (4.2%) with central nervous system involvement. 

In 67 (27.9%) patients, GI disease was confirmed with the positive endoscopic study, while 28 (11.7%) patients had suggestive symptoms but a negative endoscopic study, and GI disease was unsuspected in the remaining 145 (60.4%) patients.

### 3.2. Risk Factors for GI Involvement

Patients with GI involvement were more likely to use tobacco and to have *ENG* mutation, lower hemoglobin values at diagnosis, lower minimal hemoglobin levels during follow-up, lower ferritin levels (<15 ug/L), and higher systolic pulmonary artery pressure (sPAP) at TTE than those with no GI involvement. Compared to patients with unsuspected GI involvement, those with GI involvement were older and were more likely to use tobacco or alcohol and to have more comorbidities, higher ESS, lower ferritin (<15 ug/L), minimal hemoglobin levels, and a higher cardiac index (CI) and sPAP at TTE (Table 1).

After multivariate analysis, age (OR 1.07, 1.06–1.14, *p* = 0.033), *ENG* mutations (OR 5.7, 1.02–31.93 95% CI, *p* = 0.047), previous/current tobacco use (OR 7.8, 1.37–44.52 95% CI, *p* = 0.020), and hemoglobin values (OR 0.96, 0.93–0.96 95% CI, *p* = 0.033) were associated with GI involvement at endoscopy tests (Table 2). The ROC analysis showed a good predictive power of the associated risk factors for GI involvement (AUC = 0.834).

### 3.3. GI Involvement

Telangiectasia was more frequently found in the stomach and duodenum. All but one patient with telangiectasia based on the esophagogastroduodenoscopy (EGD) also showed telangiectases in the colonoscopy (CS). Most patients (81.5%) with gastric or duodenal telangiectasia in EGD also had small intestine involvement at VCE. The size of telangiectases most commonly was ≤3 mm in all locations, and most patients had ≤10 telangiectases per location. Multiple (>10) telangiectases were mostly found in the jejunum and ileum. 

Most patients with GI disease had hemoglobin levels < 8 g/dL and/or RBC transfusion requirements during follow-up (*n* = 44; 65.7%). These patients were older and with a higher ESS than the subgroup of patients without these conditions. No gender, epistaxis, age, or visceral involvement differences were found between any subgroups. Patients with hemoglobin levels < 8 g/dL and/or transfusion requirements had larger telangiectases (>3 mm) and needed APC therapy more often than the remaining patients with GI involvement, with no other differences in location or the number of telangiectases (Table 3).

### 3.4. Outcomes

Overall, mean follow-up was 34.2 ± 22.8 (1–124) months, with no differences between groups according to GI involvement. Patients with GI involvement required significantly more often RBC transfusions, ED attention, and hospital admissions than patients without GI disease or than those with unsuspected GI involvement. Overall, five patients (2.1%) died, with no differences in mortality between groups (Table 4). 

Nine patients received treatment with octreotide: four with 100 mcg bid and five with long-acting release (LAR) octreotide at doses between 10 and 30 mcg monthly. In five of these nine patients, a marked decrease in the number of packed RBC units transfused was observed in the first weeks of treatment. Only one patient presented diarrhea as a side effect with the 100 mcg bid dose, which disappeared after switching to a monthly LAR formulation. Two patients without improvement after octreotide daily doses received bevazicumab at doses of 5 mg/kg every two weeks with tapering frequency to a final maintenance dose every 6–8 weeks. One of these patients needed hypertension therapy adjustment because of bevacizumab-induced hypertension with severe epistaxis and an increase of RBC transfusion requirements during the first six months of treatment. After blood pressure control, both patients experienced a rather marked reduction in the number of packed RBC units transfused under bevacizumab therapy (Table 5).

## 4. Discussion

In our study, age, *ENG* mutation, tobacco use, and hemoglobin levels were associated with GI involvement in HHT patients. We found a 7% increased risk of GI involvement per year of age. These results are consistent with the mean age of GI bleeding onset observed in previous studies [4,13]. However, we have not detected previously described female predominance [4,17,21]. Smoking history has been related to a 7-fold increase risk of GI involvement in our series. Tobacco use has already been associated with an increased risk of upper GI bleeding and gastroduodenal ulcers in non-HHT patients, but no relationship between tobacco and GI telangiectasia has been published [27]. Though this finding strengthens the importance of avoiding tobacco in HHT patients, it also needs to be confirmed in further studies. Canzonieri et al. and van Tuyl et al. systemically studied the extent of GI involvement with EGD, VCE, and CS in 22 and 35 HHT patients, respectively, and found a higher prevalence of telangiectasia in patients with *ENG* mutation [13,19]. However, Berg et al., Sabbà et al., and Letteboer et al. assessing genotype–phenotype relationships in HHT patients, reported similar incidence of GI telangiectasia between HHT1 and HHT2 patients [28,29,30]. Differently, and according to current guidelines, we studied patients with clinical suspicion of GI bleeding, but not indiscriminately screened [4]. Thus, *ENG* mutation was associated with 5-fold increase risk of GI involvement compared to those with negative endoscopic study. In addition, in our cohort, hemoglobin levels were also associated with GI involvement. These four variables could help physicians experiencing the difficult management of GI involvement in HHT patients.

Many cases of anemia are misattributed to overt epistaxis instead of attributing them to GI bleeding among patients with HHT [16,31]. In fact, we have not found significant differences in epistaxis severity measured by the ESS between patients with and without GI involvement. This finding supports that lower hemoglobin levels found in patients with GI involvement were disproportionate. Moreover, among patients with GI involvement, the ESS was higher in those with hemoglobin levels < 8 g/dL or with transfusion requirements. This relationship could be explained by a microvessel predominant pattern, as telangiectasis is a pathological feature in both nasal and digestive mucosae. Because both types of bleeding can coexist, a high clinical suspicion of GI bleeding in patients with severe anemia, despite a high ESS, is necessary.

In our series, telangiectasia is more frequently found in the stomach or duodenum. This location is highly related to small bowels and colon telangiectasia in VCE. These results are in line with international guidelines, which recommend EGD as the initial screening procedure when GI involvement is suspected [4,13]. However, if the EGD is negative, VCE should be considered in patients with high suspicion of GI involvement and severe anemia to detect telangiectasia within the small intestine, as it occurred in 18.5% of our patients [13,19]. Longrace et al., in 43 consecutive HHT patients with GI bleeding, reported that patients with >20 telangiectases had significantly lower hemoglobin levels and higher transfusion requirements [21]. We found that patients with hemoglobin levels <8 g/dL or RBC transfusion requirements had larger telangiectases. These endoscopic findings strengthen the usefulness and benefits of VCE and should be taken into account in the follow-up and treatment assessment of HHT patients with GI involvement.

Chronic GI bleeding treatment is largely endoscopic and supported by frequent blood transfusions. In our study, patients with GI involvement needed RBC transfusions and medical attention more often than those with no or unsuspected GI involvement. However, no difference in mortality was found between groups. Although severe anemia secondary to GI bleeding can lead to multiple complications, GI bleeding has not been described as a cause of the lower life expectancy of HHT patients [29,32]. HHT patients with GI bleeding usually have telangiectases that are not fit for APC treatment, and require pharmacological treatment, with different and controversial options [4,13,16,21]. Our series included nine patients on octreotide treatment from daily clinical practice. This agent has shown a reduction in digestive bleeding and an anemia improvement in non-HHT patients with intestinal angiodysplasia by reducing splanchnic blood flow, but evidence of its use in HHT patients is scarce [33,34,35,36]. A non-randomized prospective clinical trial to assess the efficacy of monthly injection of 20 mg of octreotide LAR has been recently published [36]. Although this study was underpowered (beta of 0.5), RBC transfusion requirements were lower during the six months treatment period than they were prior to treatment, in all 11 patients included. We have observed similar results in five out of nine patients treated with octreotide. Further studies are needed to confirm the efficacy and safety of long-term octreotide therapy. Similarly, the benefit of bevacizumab in patients with GI involvement needs has been poorly described [37,38]. Bevacizumab-induced hypertension is a well-known adverse effect; high blood pressure can make epistaxis worse and provoke RBC transfusion requirements, as it occurred in one of our patients [39]. Other treatments such as talidomide or estrogen/progesterone preparations have also shown improvement in hemoglobin levels or transfusion requirements in case reports or short series [21,22,40,41]. The hypothetical benefit in this scenario of future agents that block or activate pathways involved in HHT pathogenesis, such as sirolimus, tacrolimus, nintedanib, or a combination of them, needs further investigation [42,43,44]. 

There are some limitations and strengths of our study that should be mentioned. VCE was not performed in all patients and GI involvement could be underestimated in patients with negative EGD. Additionally, VCE could have influenced the number and size of small bowel telangiectases. Another limitation is that endoscopic study was not performed on all patients. However, this is in agreement with recommendations of current guidelines [4]. Difficulties in attributing low hemoglobin levels to epistaxis or GI bleeding could be another inherent limitation. However, our study represents the largest series of HHT patients with objectively confirmed GI involvement, and is the only one comparing these patients with those with a negative endoscopic study or with unsuspected GI involvement. On top of that, the prospective nature of our study and the broad long-term follow-up reinforce the robustness of the results and allow for a better assessment of outcomes.

## 5. Conclusions

In conclusion, age, *ENG* mutation, tobacco use, and hemoglobin levels have been associated with GI involvement in HHT patients. Telangiectasia was more frequently found in the stomach and duodenum. Patients with large telangiectases have severe anemia and high RBC transfusion requirements. VCE should be considered in patients with a high suspicion of GI bleeding, even if EGD resulted negative. Among HHT patients, those with GI involvement need medical attention more often, with no differences in mortality.

## Figures and Tables

**Table 1:** Clinical characteristics and screening tests according to gastrointestinal involvement in 240 patients with hereditary hemorrhagic telangiectasia (HHT).

	GI Involvement (*n* = 67)	No GI Involvement (*n* = 28)	*p* Value	Unsuspected GI Involvement (*n* = 145)	*p* Value *
Clinical characteristics					
Gender (female), *n* (%)	35 (52.2)	18 (64.3)	0.380	84 (57.9)	0.437
Age years-old, mean (SD)	59.5 (11.1)	56.9 (13.3)	0.332	44.3 (16.3)	<0.001
Epistaxis age of presentation, mean (SD)	16.6 (13.1)	13.8 (7.4)	0.207	16.1 (11.9)	0.813
Underlying conditions, *n (%)*					
Smoking history	38 (56.7)	8 (28.6)	0.012	59 (40.7)	0.029
Alcoholism	15 (22.4)	4 (14.3)	0.368	13 (9)	0.007
Hypertension	25 (37.3)	11 (39.3)	0.857	27 (18.6)	0.003
Diabetes mellitus	14 (20.9)	3 (10.7)	0.238	9 (6.2)	0.001
Dislipemia	24 (35.8)	11 (39.3)	0.750	31 (21.4)	0.026
Ischemic heart disease	15 (22.4)	3 (10.7)	0.186	12 (8.3)	0.004
Lung disease	19 (28.4)	6 (21.4)	0.484	12 (8.3)	<0.001
CNS ischemic disease	13 (19.4)	1 (3.6)	0.059	11 (7.6)	0.012
Heart failure	7 (10.4)	0	0.101	2 (1.4)	0.005
Cancer	9 (13.4)	0	0.054	8 (5.5)	0.048
Atrial fibrillation	9 (13.4)	2 (7.1)	0.498	4 (2.8)	0.005
HHT screening,					
Curaçao criteria ≥ 3, *n* (%)	67 (100)	27 (96.4)	0.295	136 (93.8)	0.060
Epistaxis, *n* (%) ESS, mean (SD) ESS ≥ 4, *n* (%)	67 (100) 4.2 (2.2) 35 (56.5)	27 (96.4) 3.5 (1.9) 10 (37)	0.295 0.137 0.092	137 (94.5) 3.4 (2.2) 52 (36.4)	0.058 0.019 0.009
Family history, n (%)	61 (91)	26 (92.9)	1.000	141 (97.2)	0.076
Muco-cutaneous telangiectasia, n (%)	67 (100)	27 (96.4)	0.295	139 (95.9)	0.180
Visceral involvement (excluding GI involvement), *n* (%)	53 (79.1)	19 (67.9)	0.318	89 (61.3)	0.012
Genetic test, *n* (%)					
Undergone	40 (59.7)	20 (71.4)	0.280	106 (73.1)	0.050
ENG	21 (52.5)	4 (20)	0.001	50 (49)	0.709
ACVRL1	17 (42.5)	14 (70)	0.044	42 (41.2)	0.886
Negative	2 (5)	2 (10)	0.595	9 (8.8)	0.728
Blood test,					
Hemoglobin levels (g/dL), mean (SD)	113.1 (24.7)	131.4 (22.6)	0.007	133.9 (22.9)	<0.001
Minimal hemoglobin levels (g/dL), mean (SD)	84.3 (30.8)	108.3 (32.7)	0.002	118.2 (30.5)	<0.001
Ferritin level < 15 ug/L, n (%)	56 (83.6)	15 (55.6)	0.004	76 (55.1)	<0.001
Other tests,					
Positive contrast TTE, *n* (%)	45 (67.1)	13 (46.4)	0.252	94 (64.8)	0.640
CI at TTE (l/min/m²), mean (SD)	3.4 (0.9)	3.4 (1.2)	0.988	2.9 (0.6)	<0.001
sPAP at TTE (mm Hg), mean (SD)	37.9 (12.3)	31.3 (7.1)	0.024	29.8 (6.9)	<0.001
sPAP > 40mmHg, *n* (%)	12 (32.4)	2 (15.4)	0.303	6 (9.5)	0.004
Pulmonary AVM at CT, *n* (%)	25 (37.3)	3 (10.7)	0.256	39 (26.8)	0.077
Abdominal CT, *n* (%)	62 (92.5)	22 (78.6)	0.077	103 (71)	<0.001
Hepatic involvement	40 (59.7)	17 (60.7)	0.271	53 (36.5)	0.101
Other visceral involvement	34 (50.7)	10 (35.7)	0.487	29 (20)	0.001
CNS involvement, *n* (%)	5 (7.5)	0	0.298	5 (3.4)	0.482

GI: Gastrointestinal; ACVRL1: Activin A receptor type II-like 1 gene; ENG: Endoglin gene; SD: Standard deviation; CI: Cardiac index; sPAP: Systolic pulmonary artery pressure; TTE: Transthoracic echocardiography; CNS: Central nervous system. * Comparison between GI involvement and unsuspected GI involvement groups.

**Table 2:** Uni- and multivariable logistic regression analyses for gastrointestinal bleeding in patients with HHT.

	Univariable			Multivariable		
	OR	95% CI	*p*	OR	95% CI	*p*
Male	1.64	0.66–4.08	0.283	0.90	0.15–5.32	0.910
Age, years	1.02	0.98–1.05	0.339	1.07	1.06–1.14	0.033
Age > 50 years	2.30	0.86–6.16	0.095	2.06	0.07–61.42	0.676
Smoking	3.27	1.26–8.48	0.015	7.82	1.37–44.52	0.020
*ENG* mutations	4.42	1.25–15.57	0.021	5.72	1.02–31.93	0.047
*ACVRL1* mutations	0.31	0.10–0.99	0.049	1.99	0.07–54.84	0.685
Ferritin levels < 15 ug/L	4.07	1.5–11.03	0.006	3.09	0.55–17.5	0.202
Hemoglobin, g/dL	0.97	0.95–0.99	0.003	0.96	0.93–0.96	0.033
Hemoglobin levels < 8 g/dL	2.18	0.83–5.76	0.112	0.75	0.09–6.55	0.801
ESS ≥ 4	2.20	0.87–5.57	0.095	1.57	0.26–9.66	0.624

OR: odds ratio; 95% CI: 95% confidence intervals; *ACVRL1*: activin A receptor type II-like 1 gene; *ENG*: endoglin gene; ESS: epistaxis severity score.

**Table 3:** Clinical characteristics and endoscopic findings among patients with gastrointestinal involvement.

	Patients with Hb ≤ 8 or Transfusion (*n* = 44)	Patients with Hb > 8 and No Transfusion (*n* = 23)	*p* Value
Clinical characteristics			
Gender (female), *n* (%)	22 (50)	13 (56.5)	0.612
Age at presentation, mean (SD)	61.3 (11.2)	53.8 (9.6)	0.006
Epistaxis age at presentation, mean (SD)	16.5 (12.9)	16.7 (13.7)	0.941
ESS, mean (SD) ESS > 4, *n* (%)	4.9 (2.2) 27 (69.2)	2.9 (1.6) 8 (34.8)	<0.001 0.008
Visceral involvement (excluding *GI* involvement), *n* (%)	34 (77.3)	18 (78.3)	0.927
GI tests, *n* (%)			
Both EGD and CS	30 (68.2)	13 (56.5)	0.345
Video capsule endoscopy	20 (45.5)	7 (30.4)	0.234
APC therapy	25 (64.1)	6 (30%)	0.013
Site of GI telangiectases, *n* (%)			
Esophagical >10 telangiectases Size: ≤3 mm >3 mm	7 (15.9) 0 4 (66.7) 2 (33.3)	3 (13) 0 3 (100) 0	1.000 0.500
Gastric >10 telangiectases Size: ≤3 mm >3 mm	36 (81.8) 6 (16.7) 12 (42.9) 16 (57.1)	16 (69.6) 2 (12.5) 11 (73.3) 4 (26.7%)	0.253 1.000 0.056
Duodenal >10 telangiectases Size: ≤3 mm >3 mm	31 (70.5) 6 (19.4) 15 (55.6) 12 (44.4)	13 (56.5) 1 (7.1) 10 (83.3) 2 (16.7)	0.254 0.407 0.151
Jejunal >10 telangiectases (n) Size: ≤3 mm >3 mm	21 (47.7) 10 (47.6) 10 (50) 10 (50)	7 (30.4) 3 (42.9) 6 (85.7) 1 (14.3)	0.173 1.000 0.183
Ileal >10 telangiectases Size: ≤3mm >3 mm	15 (75) 8 (57.1) 5 (55.6) 4 (44.4)	5 (71.4) 1 (20) 4 (100) 0	0.294 0.303 0.228
Colonic >10 telangiectases Size: ≤3 mm >3 mm	16 (39) 1 (6.7) 7 (58.3) 5 (41.7)	5 (23.8) 0 2 (66.7) 1 (33.3)	0.231 1.000 1.000
Patients with large telangiectases (>3 mm) (all tests), *n* (%)	23 (65.7)	3 (20)	0.003
Patients with >10 telangiectases in any location (all tests), *n* (%)	20 (46.5)	7 (35)	0.390

APC: argon plasma coagulation; EGD: esophagogastroduodenoscopy; EES: epistaxis severity score; CS: colonoscopy; *GI*: gastrointestinal; Hb: Hemoglobin; SD: standard deviation.

**Table 4:** Clinical outcomes during follow-up according to gastrointestinal involvement.

	GI Involvement (n = 67)	No GI Involvement (*n* = 28)	*p* Value	Unsuspected GI Involvement (*n* = 145)	*p* Value *
Follow-up (months), mean (SD)	33.6 (21.9)	29.2 (20.5)	0.354	35.4 (23.6)	0.585
Outcomes,					
RBC transfusion, *n* (%) Number of transfusions, mean (range)	41 (61.2) 26.2 (1–218)	8 (28.6) 8.9 (2–35)	0.004 0.042	22 (15.4) 9.1 (1–50)	<0.001 0.101
ED visit, *n* (%)	40 (59.7)	7 (25)	0.002	42 (29)	<0.001
Hospital admission, *n* (%)	28 (41.8)	5 (17.9)	0.025	16 (11)	<0.001
Mortality, *n* (%)	2 (3%)	0	1.000	3 (2.1)	0.652

GI: gastrointestinal; SD: standard deviation; RBC: red blood cells; ED: Emergency Department; * Comparison between GI involvement and unsuspected GI involvement groups.

**Table 5:** Evolution of patients with gastrointestinal involvement treated with octreotide and bevacizumab.

Patients	Gender	Age (Years)	VCE	Large (>3 mm) Telangiectases	>10 Telangiectases	Site	Before Treatment		Octreotide			Bevacizumab	
							Follow-Up (m)	nº RBC	Follow-Up (m)	nº RBC	Dose	Follow-Up (m)	nº RBC
P.1	Male	68	Yes	Yes	No	G-D-J	66	11	21	4	10 mcg/m	-	-
P.2	Male	71	Yes	Yes	No	G-D	8	22	12	0	100 mcg/12 h	-	-
P.3	Male	55	Yes	Yes	No	G-D	38	8	3	1	20 mcg/m	-	-
P.4	Male	58	No	No	No	G-D	40	20	2	0	100 mcg/12 h	-	-
P.5	Female	55	No	Yes	Yes	G-D-J-I	21	9	16	3	30 mcg/m	-	-
P.6	Male	66	Yes	No	Yes	G-D-J-I	100	>100	15	92	100 mcg/12 h	21	3
P.7	Male	51	Yes	Yes	Yes	G-D-J-I	74	93	6	12	100 mcg/12 h	13	13 *
P.8	Female	51	Yes	Yes	Yes	G-D-J-I	36	36	9	5	10 mcg/m	-	-
P.9	Male	62	Yes	Yes	Yes	G-D-J-I	48	48	4	0	20 mcg/m	-	-

VCE: video capsule endoscopy; G: Gastric; D: Duodenal; I: ileal; J: jejunal; m: month; nº RBC: number of units of packed red blood cells transfused before and during octreotide or bevacizumab treatments. * All during the first six months of bevacizumab treatment because of bevacizumab-induced hypertension and resulting severe epistaxis.
